# Microbiome dysbiosis in SARS-CoV-2 infection: implication for pathophysiology and management strategies of COVID-19

**DOI:** 10.3389/fcimb.2025.1537456

**Published:** 2025-04-22

**Authors:** Shukur Wasman Smail, Niaz Albarzinji, Rebaz Hamza Salih, Kalthum Othman Taha, Sarah Mousa Hirmiz, Hero M. Ismael, Marwa Fateh Noori, Sarkar Sardar Azeez, Christer Janson

**Affiliations:** ^1^ College of Pharmacy, Cihan University-Erbil, Erbil, Kurdistan Region, Iraq; ^2^ Department of Biology, College of Science, Salahaddin University-Erbil, Erbil, Kurdistan Region, Iraq; ^3^ College of Medicine, Hawler Medical University, Erbil, Iraq; ^4^ Department of Respiratory Medicine, PAR Private Hospital, Erbil, Kurdistan Region, Iraq; ^5^ Department of Medical Laboratory Technology, Soran Technical College, Erbil Polytechnic University, Erbil, Kurdistan Region, Iraq; ^6^ Department of Medical Science, Respiratory Medicine, and Allergology, Uppsala University and University Hospital, Uppsala, Sweden

**Keywords:** COVID-19, SARS-CoV-2, gut microbiota, dysbiosis, prognosis, diagnosis, therapeutics

## Abstract

The emergence of severe acute respiratory syndrome coronavirus 2 (SARS-CoV-2), the etiological agent of coronavirus disease 2019 (COVID-19), in late 2019 initiated a global health crisis marked by widespread infection, significant mortality, and long-term health implications. While SARS-CoV-2 primarily targets the respiratory system, recent findings indicate that it also significantly disrupts the human microbiome, particularly the gut microbiota, contributing to disease severity, systemic inflammation, immune dysregulation, and increased susceptibility to secondary infections and chronic conditions. Dysbiosis, or microbial imbalance, exacerbates the clinical outcomes of COVID-19 and has been linked to long-COVID, a condition affecting a significant proportion of survivors and manifesting with over 200 symptoms across multiple organ systems. Despite the growing recognition of microbiome alterations in COVID-19, the precise mechanisms by which SARS-CoV-2 interacts with the microbiome and influences disease progression remain poorly understood. This narrative review investigates the impact of SARS-CoV-2 on host-microbiota dynamics and evaluates its implications in disease severity and for developing personalized therapeutic strategies for COVID-19. Furthermore, it highlights the dual role of the microbiome in modulating disease progression, and as a promising target for advancing diagnostic, prognostic, and therapeutic approaches in managing COVID-19.

## 1 Introduction

The human microbiome constitutes a diverse and complex ecosystem of microorganisms that play a critical role in health and physiological function (Ogunrinola et al., 2020). Comprising bacteria, archaea, eukaryotes, and viruses, these microbial communities inhabit distinct niches within the body (Mousa et al., 2022). While some microbes exhibit pathogenic potential, many are indispensable, facilitating digestion, regulating metabolism, enhancing immune defence, and preventing pathogen colonization through competitive exclusion (Pickard et al., 2017).

Recently, it’s been found that Severe Acute Respiratory Syndrome Coronavirus 2 (SARS-CoV-2) infection disrupts the gut microbiome, leading to dysbiosis that exacerbates disease severity and contributes to systemic inflammation, immune dysregulation, and increased susceptibility to secondary infections and chronic conditions (Raj et al., 2024). The coronavirus disease 2019 (COVID-19) caused by SARS-CoV-2, has emerged as a global pandemic, surpassing previous coronavirus outbreaks, such as severe acute respiratory syndrome (SARS) and Middle East respiratory syndrome (MERS), in scale and impact. Since its initial identification in Wuhan, China, in late 2019, COVID-19 has primarily affected the respiratory system and has resulted in over six million deaths worldwide (Lai et al., 2020; Cascella et al., 2024). Globally, COVID-19 has triggered an acute health crisis and widespread socio-economic disruption, mental health deterioration, and setbacks in medical care, all of which have contributed to increased mortality. The pandemic has exacerbated educational inequalities, heightened psychological distress, and led to a rise in self-harm and suicide rates. Furthermore, long-term COVID-19, affecting at least 10% of infected individuals, manifests with over 200 symptoms across multiple organ systems, impairing daily function and delaying workforce reintegration. Currently, no standardized treatment exists for this condition (Zhu et al., 2023).

The interplay between the human microbiome and host immune function is well established, with microbial homeostasis playing a crucial role in maintaining immune tolerance, metabolic regulation, and resistance to infections (Zheng et al., 2020). Despite the growing recognition of microbiome disturbances in viral infections, the precise mechanisms by which SARS-CoV-2 disrupts microbial communities and contributes to systemic inflammation, immune dysregulation, and post-acute sequelae (long-COVID) remain unclear. Current research primarily focuses on the direct viral effects on the respiratory system, overlooking the broader implications of microbiome alterations on disease progression, host resilience, and secondary complications (Kopańska et al., 2022).

Moreover, while microbiome-targeted interventions such as probiotics, prebiotics, and faecal microbiota transplantation (FMT) have shown promise in modulating immune responses and restoring microbial balance, their potential role in mitigating COVID-19 severity and long-term consequences remains underexplored (Abavisani et al., 2025). Addressing these gaps is crucial for understanding the bidirectional relationship between SARS-CoV-2 and the microbiome, identifying microbial biomarkers for disease prognosis, and developing targeted microbiome therapies to improve patient outcomes in acute and long-term COVID-19 cases.

This narrative review explores the role of gut microbiome dysbiosis in COVID-19 pathogenesis and its implications in the post-acute phase. It also investigates the interaction between the host microbiome and SARS-CoV-2, highlighting its potential for novel diagnostic strategies, prognostic indicators, and the therapeutic potential of microbiome modulation in preventing and mitigating COVID-19 complications.

## 2 Gut microbiome dysbiosis in COVID-19

SARS-CoV-2 infection induces profound alterations in the gut microbiome, leading to a state of dysbiosis, characterized by microbial imbalance, reduced diversity, and an overrepresentation of pathogenic species (Ancona et al., 2023). Recent studies have highlighted a decline in beneficial commensal bacteria, such as *Faecalibacterium prausnitzii* and *Bifidobacterium* spp., alongside an increase in opportunistic pathogens, including *Enterococcus* and *Escherichia coli*, in COVID-19 patients (Wang et al., 2023; Zhang et al., 2023). This shift is associated with heightened inflammation, impaired gut barrier function, and systemic immune activation, further exacerbating disease severity and post-acute complications (Yousef et al., 2024). Several mechanisms have been proposed to explain how SARS-CoV-2 infection disrupts the gut microbiome composition, leading to dysbiosis in the following sub-sections.

### 2.1 SARS-CoV2-induced apoptosis of enterocytes and impaired gut barrier function

Enterocytes, the absorptive epithelial cells lining the small intestine, play a pivotal role in maintaining gut homeostasis through nutrient absorption, barrier integrity, immune regulation, and interaction with the gut microbiome (Takiishi et al., 2017). They facilitate the uptake of amino acids, lipids, vitamins, and carbohydrates through specialized transporters (Kiela and Ghishan, 2016). Their barrier function, maintained by tight junction proteins like claudins, occludin, and ZO-1, prevents pathogen translocation into the bloodstream (Hartsock and Nelson, 2008; Di Tommaso et al., 2021). Additionally, enterocytes regulate gut microbiota by producing antimicrobial peptides that sustain microbial balance and immune homeostasis (Ra and Bang, 2024). Disruptions due to infection or inflammation can impair their function, leading to gut dysbiosis, increased permeability (leaky gut), and systemic inflammation (Zheng et al., 2020).

Several studies have provided direct evidence that SARS-CoV-2 can infect enterocytes, contributing to gut dysbiosis and systemic inflammation (Lamers et al., 2020). Studies utilizing human small intestinal enteroids confirmed that SARS-CoV-2 productively infects gut enterocytes (Lamers et al., 2020). Furthermore, investigations have shown that viral infection of enterocytes induces a strong inflammatory response, disrupting intestinal homeostasis and contributing to the gastrointestinal symptoms seen in COVID-19 patients (Villapol, 2020). SARS-CoV-2 enters intestinal enterocytes primarily by binding its spike (S) protein to the ACE2 receptor, which is abundantly expressed on the surface of enterocytes (Zamorano Cuervo and Grandvaux, 2020; Zhang et al., 2020). After binding, the transmembrane serine protease 2 (TMPRSS2) facilitates the viral entry by cleaving the S protein, enabling the virus to fuse with the host cell membrane, followed by viral replication within the gut (Hoffmann et al., 2020). Once inside the host cell, the viral RNA is translated into proteins, and the viral genome replication begins (Lou and Rao, 2022). The formation of replication-transcription complexes (RTCs) allows the virus to hijack the host’s machinery to produce viral particles, releasing new virions from the infected enterocytes, which can infect neighbouring cells and spread throughout the gut (Malone et al., 2022).

This process is compounded by viral-induced inflammation and the activation of PRRs, including TLRs, which sense viral components and trigger inflammatory responses and the heightened secretion of pro-inflammatory molecules such as IL-6, TNF-α, and IFNS, which can lead to the downregulation of these tight junctions in nearby cells (Cao et al., 2013; Devaux et al., 2021; Gou et al., 2021). This inflammatory milieu recruits immune cells to the site of infection, intensifying the immune response and exacerbating local tissue damage​ (Merad et al., 2022). The disruption in barrier integrity causes a leaky gut, where pathogens and toxins can pass into the bloodstream, leading to systemic inflammation and complications such as endotoxemia and multi-organ dysfunction (Kim, 2021). Certain commensals, particularly those belonging to the Bacteroidetes phylum, involving *Bacteroides ovatus, Bacteroides dorei, Bacteroides massiliensis*, and *Bacteroides thetaiotaomicron*, appear to interact inversely with SARS-CoV-2 concentrations in faecal specimens. Such bacteria are thought to play a role in suppressing ACE2 receptors in intestinal regions (Zuo et al., 2020; Wang et al., 2023).

### 2.2 ACE2 depletion and the disruption of the renin-angiotensin aldosterone system

ACE2 is a membrane-bound enzyme that regulates both the RAAS and gut homeostasis. In the intestines, it is highly expressed on enterocytes, where it facilitates amino acid absorption, particularly tryptophan, which influences serotonin synthesis, antimicrobial peptide production, and gut microbiota composition (Camargo et al., 2020; Khemaissa et al., 2022). Additionally, ACE2 counterbalances the effects of angiotensin II (Ang II), a pro-inflammatory mediator implicated in hypertension and systemic inflammation (Marchesi et al., 2008; Di Raimondo et al., 2012). By converting Ang II into Ang- (Pickard et al., 2017; Lai et al., 2020; Ogunrinola et al., 2020; Mousa et al., 2022; Zhu et al., 2023; Cascella et al., 2024; Raj et al., 2024), ACE2 promotes vasodilation, reduces inflammation, and maintains epithelial integrity (Sampaio et al., 2007; Bader, 2013; Li et al., 2022; Chittimalli et al., 2023). Changes in the transport of amino acids alter the production of several beneficial metabolites such as SCFA, butyrate, propionic acid, and acetic acid, subsequently altering the balance of gut microbiota (Clerbaux et al., 2022). Moreover, a decrease in antimicrobial peptide production also contributes to dysbiosis, allowing the overgrowth of pathogenic bacteria in the gut (Hashimoto et al., 2012).

SARS-CoV-2 binding to ACE2 leads to internalization and downregulation of ACE2 receptors in enterocytes (Banu et al., 2020; Lu et al., 2022). In a study, ACE2 and B0AT1-knockout (KO) mice demonstrated decreased serum tryptophan levels and disrupted intestinal production of antimicrobial peptides, which was reversed upon tryptophan supplementation (Battagello et al., 2020). Several other studies have shown ACE2 protein and mRNA level dysregulation in different cell and organoid systems, with some conflicting findings (Lamers et al., 2020; Glowacka et al., 2009; Nataf and Pays, 2021). This downregulation impairs the uptake of essential amino acids like tryptophan, affecting serotonin production and antimicrobial peptide synthesis, which are vital for maintaining gut microbial balance (Qin et al., 2021). These studies show that perturbed tryptophan and amino acid transport deteriorates the production of beneficial products and adversely alters gut microbiota.

ACE2 is a key regulator of the RAAS, where it balances the actions of Ang II and converts it to Ang (Pickard et al., 2017; Lai et al., 2020; Ogunrinola et al., 2020; Mousa et al., 2022; Zhu et al., 2023; Cascella et al., 2024; Raj et al., 2024), which has anti-inflammatory and vasodilatory effects, thus protecting tissues from damage, maintaining homeostasis and the integrity of epithelial cells (Ferrario et al., 2005; Khajah et al., 2016). SARS-CoV-2 binding to ACE2 disrupts this delicate balance, leading to a build-up of Ang II (Camargo et al., 2022). Elevated levels of Ang II promote vasoconstriction, oxidative stress, and inflammation, which can compromise intestinal barrier integrity and increase gut permeability (Chittimalli et al., 2023). This facilitates the translocation of bacterial endotoxins from the gut lumen into the bloodstream, exacerbating systemic inflammation and contributing to COVID-19 severity (Tadros et al., 2000). ACE2 loss is associated with gastrointestinal leakage and gut dysbiosis, with subsequent pulmonary hypertension (Kim et al., 2020). Moreover, elevated Ang II levels are associated with prolonged COVID-19 infection, viral load, and severity (Liu et al., 2020; Camargo et al., 2022).

**Figure 1:**
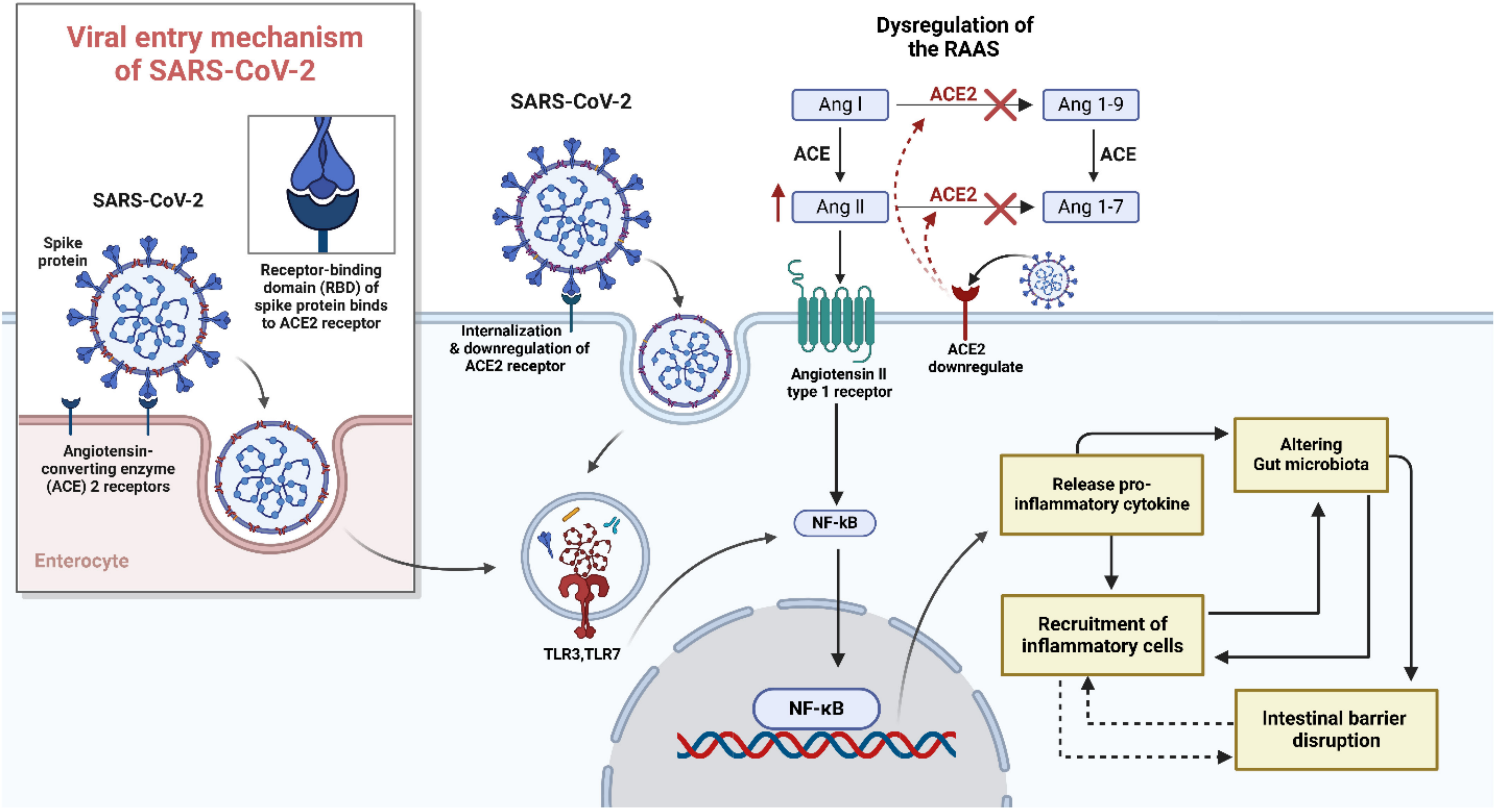
Pathological mechanism of SARS-CoV-2 and inducing gut dysbiosis. There is a potential link between SARS-CoV-2 infection and gut dysbiosis, an imbalance in the gut microbiota composition. The entry of SARS-CoV-2 into the body is mediated by the angiotensin-converting enzyme 2 (ACE2) receptor, which is highly expressed in various tissues, including the gastrointestinal tract. The downregulation of ACE2 disrupts the renin-angiotensin-aldosterone system (RAAS), leading to increased oxidative stress, inflammation, and hypertension. Once the virus gains access to the body, it primarily infects respiratory epithelial cells, leading to respiratory symptoms such as cough, shortness of breath, and pneumonia. However, emerging evidence suggests that SARS-CoV-2 can infect and replicate in the intestinal epithelial cells, resulting in gastrointestinal symptoms such as diarrhea, nausea, and vomiting. The mechanism by which SARS-CoV-2 induces gut dysbiosis involves several factors. Firstly, the direct infection of intestinal epithelial cells by SARS-CoV-2 may lead to epithelial damage and subsequent alterations in the gut microbiota composition. Secondly, the immune response triggered by SARS-CoV-2 infection, characterized by excessive inflammation through toll-like receptor (TLR) and activation of nuclear factor kappa B (NF-KB) and cytokine release, can disrupt the delicate balance of the gut microbiota.

A pivotal study using RNA sequencing of autopsy samples from SARS-CoV-2 patients found that immune and RAAS-related gene dysregulation contributed to COVID-19 severity. Upregulated genes in major organs—but not in mediastinal lymph nodes—were associated with fibrin deposition, vascular leakage, thrombosis, and mitochondrial dysfunction. Histological analysis revealed lymph node fibrosis, immune disruption, and excess collagen deposition, impairing immune response. These findings, which were also observed in animal models and human blood samples, suggested that cytokine storm in severe COVID-19 was driven by upstream immune and mitochondrial signalling, ultimately leading to RAAS overactivation and systemic organ damage (Topper et al., 2024). Thus, disruption of RAS and elevation of Ang II due to dysregulation ACE2 induced gut dysbiosis through deterioration of gut-barrier function (**Figure 1**).

## 3 Consequences of gut microbiome dysbiosis

### 3.1 Intestinal inflammation and immune dysregulation

The primary events caused by SARS-CoV-2 intestinal infection significantly contribute to gut dysbiosis, inflammation, and systemic immune dysregulation. The leaky gut barrier permits microbial products such as LPS and fungal β-glucans to enter the systemic circulation, a phenomenon known as microbial translocation, which triggers widespread inflammation (Giron et al., 2022; Brooks et al., 2024).

**Figure 2:**
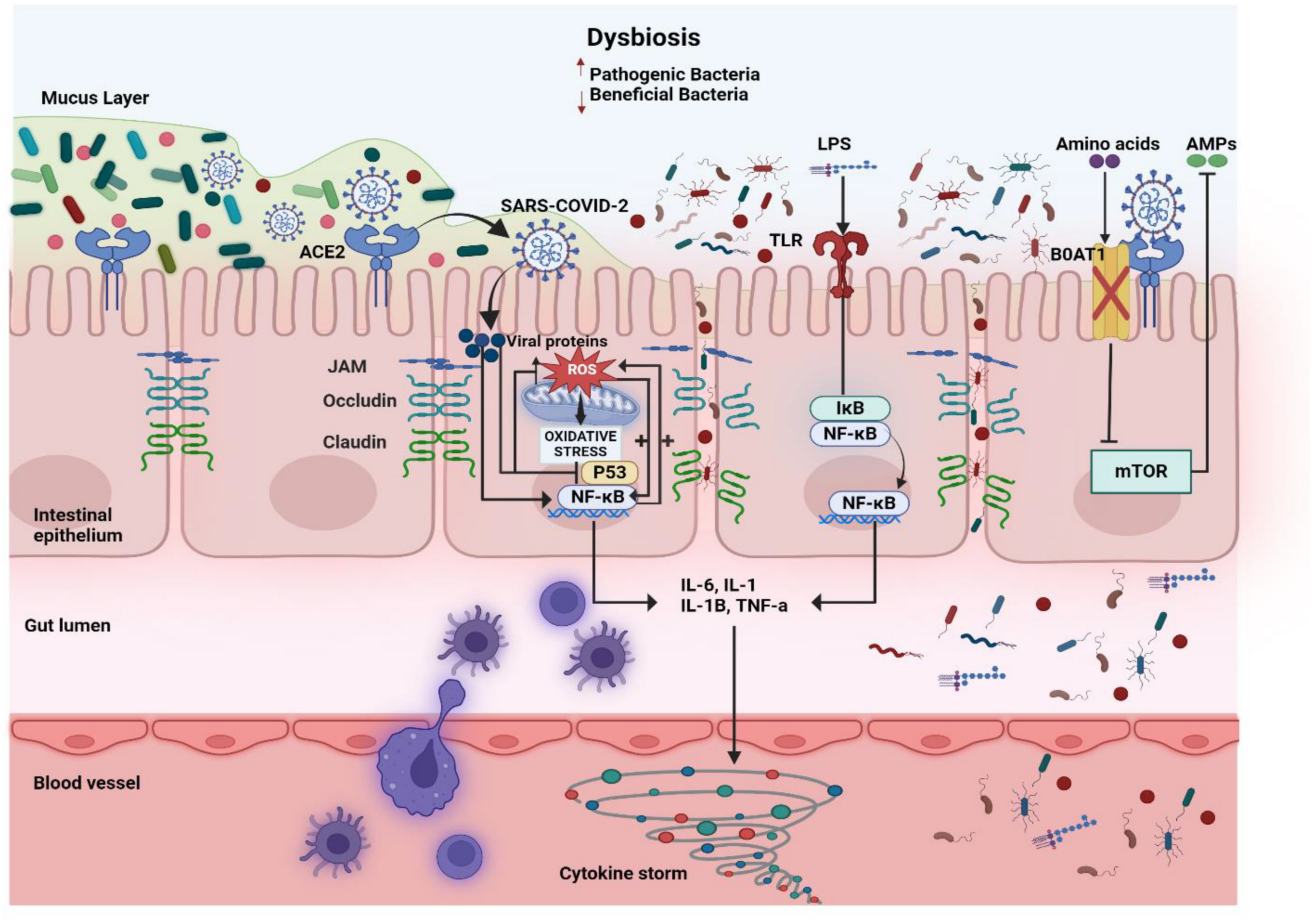
The role of gut microbiome imbalance in COVID-19. SARS-CoV-2 induces gut dysbiosis by disrupting the balance between beneficial bacteria (which protects the gut) and pathogenic bacteria (which promotes inflammation). The virus uses the Angiotensin-Converting Enzyme 2 (ACE2) receptor on intestinal epithelial cells to enter, causing downstream effects that impair gut integrity and immune function. Inside epithelial cells, viral proteins increase Reactive Oxygen Species (ROS), triggering oxidative stress and activating the transcription factors nuclear factor Kappa B Cells (NF-κB) and Tumor Protein 53 (p53). These pathways promote inflammation and cell damage. At the same time, the gut’s tight junction proteins, such as Junctional Adhesion Molecule (JAM), Occludin, and Claudin, are disrupted, leading to the breakdown of the intestinal barrier. The dysbiotic environment allows Lipopolysaccharides (LPS), a component of pathogenic bacteria, to activate Toll-Like Receptors (TLRs) on epithelial cells. This degrades Inhibitor of NF-κB (IκB), further stimulating NF-κB and resulting in the production of pro-inflammatory cytokines, such as Interleukin-6 (IL-6), IL-1, IL-1β, and tumor necrosis factor alpha (TNF-α). These cytokines contribute to a cytokine storm, exacerbating local and systemic inflammation. Additionally, SARS-CoV-2 reduces the function of B0AT1 Amino Acid Transporter (BOAT1), impairing amino acid absorption and metabolic regulation through the Mammalian Target of Rapamycin (mTOR) pathway. The reduced production of Antimicrobial Peptides (AMPs) weakens the gut’s natural defenses, allowing pathogenic bacteria to proliferate.

The GALT maintains immune surveillance and is significantly altered during SARS-CoV-2 infection. Dysbiosis shifts the balance of immune cell populations, leading to excessive activation of mucosal T helper and CTL and a decline in Tregs (Laing et al., 2020; Lehmann et al., 2021). This imbalance favours pro-inflammatory pathways, particularly the over-activation of Th17 cells, which secrete IL-17 (Weaver et al., 2013). IL-17 exacerbates local inflammation, weakens barrier integrity, and perpetuates a cycle of intestinal damage (Sun et al., 2023). Neutrophils and macrophages infiltrate the intestinal mucosa in response to microbial translocation and epithelial injury, releasing large amounts of pro-inflammatory cytokines such as IL-6, TNF-α, and IL-1β (**Figure 2**) (Raj et al., 2024). Faecal calprotectin, predominantly secreted by activated neutrophils, is notably elevated in COVID-19 patients, particularly those with severe disease (Shokri-Afra et al., 2021; Zerbato et al., 2021). COVID-19 patients exhibit marked changes in their gut microbiota, with a reduction in beneficial bacteria such as *Faecalibacterium prausnitzii*, *Eubacterium rectale*, and *Bifidobacterium adolescentis*, which are known to support immune regulation and epithelial health ​ (Zuo et al., 2020; Yeoh et al., 2021; Liu et al., 2023). At the same time, pathogenic bacteria like *Escherichia coli* and *Enterococcus faecalis* become overrepresented, further contributing to intestinal inflammation and barrier dysfunction (Zhang et al., 2023). These inflammatory processes further impair the gut’s immune barrier, creating a hyperinflammatory intestinal environment that fails to contain the virus, further disrupts the gut microbiome composition and is a source of systemic inflammation​ and perturbed gut-lung axis.

### 3.2 Gut-organ axis: implications for systemic disease and long-term outcomes

**Figure 3:**
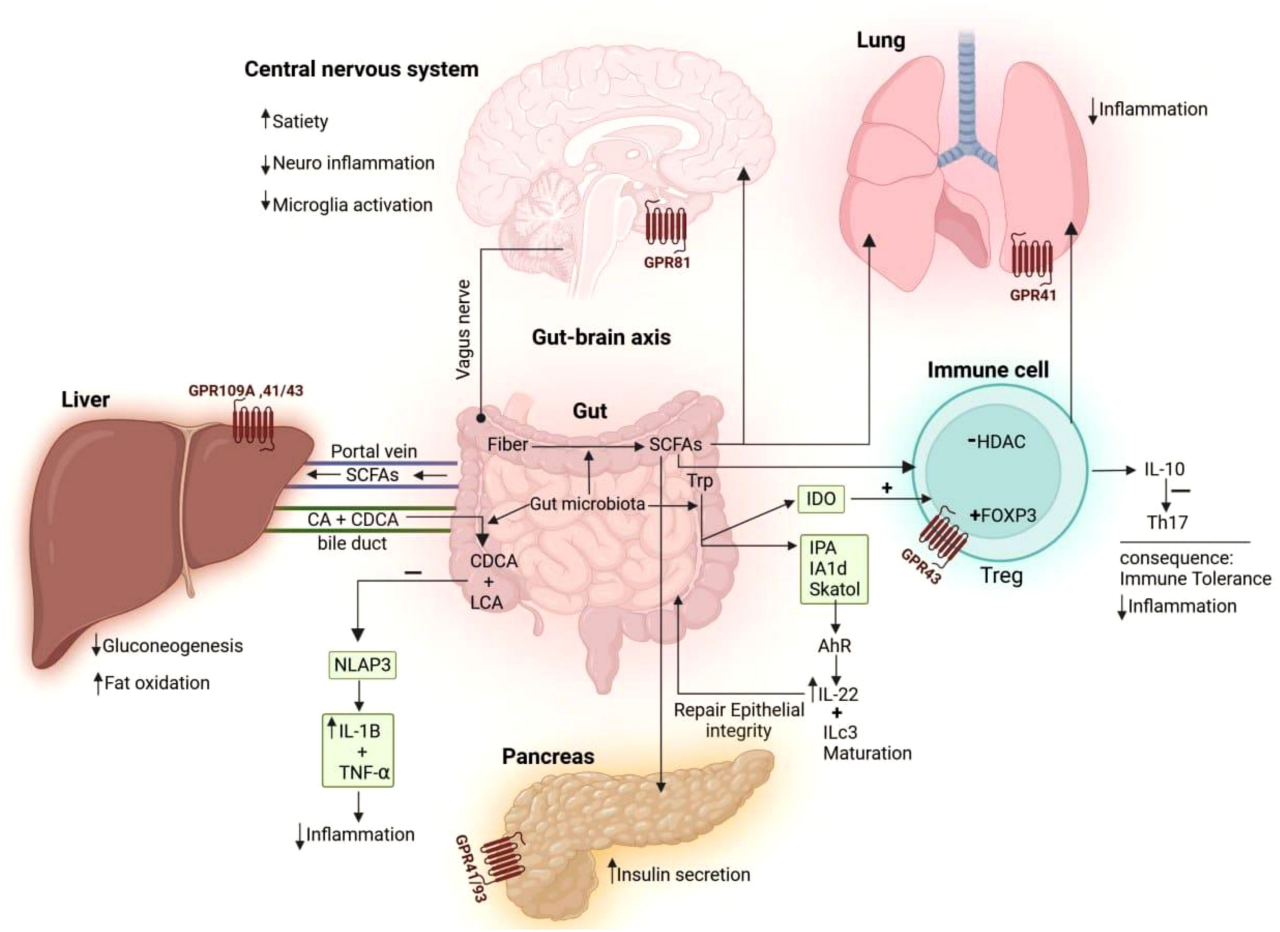
The mechanisms through which gut microbiota influence the immune system and different organs. Dietary fiber, broken down by gut bacteria, produces short-chain fatty acids (SCFAs) that act on the brain’s GPR81 receptor, boosting satiety, reducing neuroinflammation, and decreasing microglial activation. SCFAs interact with GPR43 on regulatory T cells (Tregs) to boost FOXP3 expression and hinder histone deacetylase (HDAC) activity, prompting Tregs to secrete interleukin (IL-10), which suppresses T helper 17 (Th17) cells and reduces inflammation. SCFA receptors on the lung (GPR41) and pancreas (GPR41/93) help reduce inflammation and boost insulin secretion, respectively. The gut-liver axis is influenced by SCFAs produced by the gut, which activate receptors (GPR109A, 41/43) to reduce gluconeogenesis and enhance fat oxidation. Bacteria in the gut transform primary bile acids, like cholic acid (CA) and chenodeoxycholic acid (CDCA), into secondary bile acids, acid (DCA) and lithocholic acid (LCA). These secondary bile acids then reduce inflammation by blocking NLRP3 inflammasome activity, which in turn lowers the production of pro-inflammatory cytokines, such as IL-1β and tumor necrosis factor- alpha (TNF-α). The intestinal microbiome plays a role in transferring the amino acid tryptophan (Trp), which is then converted into indoleamine 2,3-dioxygenase (IDO). IDO influences immune tolerance by promoting Treg differentiation. Trp can be transformed into indole derivatives like indole-3-propionic acid (IPA), indole-3-acetaldehyde (IAAld), and skatole. Indoles activate the aryl hydrocarbon receptor (AhR) to boost IL-22 production, which is crucial for maintaining the epithelial barrier and controlling commensal bacteria growth.

The gut–organ axis represents a complex communication network between the gut microbiota and various organs, influencing overall body homeostasis through neural, endocrine, immune, humoral, and metabolic pathways (**Figure 3**). The gut microbiota plays a crucial role in regulating physiological functions beyond digestion, impacting the brain, liver, lungs, and cardiovascular system. Disruptions in gut microbiota composition have been linked to various diseases, including neurological disorders, metabolic syndromes, and immune dysfunctions, though the precise mechanisms remain under investigation. Understanding these intricate interactions is essential for developing targeted therapeutic strategies that leverage the gut microbiome’s influence on systemic health. This review emphasizes the importance of further research into the gut–organ axis to fully comprehend how microbial communities shape human physiology and disease outcomes (Ahlawat et al., 2021).

#### 3.2.1 Gut-lung axis implications and systemic inflammation

The gut-lung axis represents a dynamic and bidirectional interplay between gastrointestinal and respiratory microbiomes, essential for maintaining immune and epithelial homeostasis in both systems (Enaud et al., 2020). This crosstalk is mediated by microbial metabolites such as SCFAs, cytokines, and immune cells circulating through the systemic bloodstream.

The immunological coordination between the gut and lungs plays a crucial role in SARS-CoV-2 infection, highlighting the interconnectedness of these two organs. The ACE2 receptor, which facilitates viral entry, is abundantly expressed in both the brush border of gut enterocytes and the alveolar epithelial type II cells in the lungs. This dual presence suggests a potential link between COVID-19 and the enteric microbiota, influencing disease severity and symptom manifestation. A significant number of COVID-19 patients exhibit gastrointestinal symptoms, with viral RNA detected in faecal samples, underscoring the need for heightened hygiene measures. Understanding this gut-lung axis is essential, particularly in inflammatory bowel disease (IBD) and non-IBD patients, as it may contribute to viral replication and systemic inflammation, affecting both respiratory and gastrointestinal health (Ahlawat et al., 2020).

**Figure 4:**
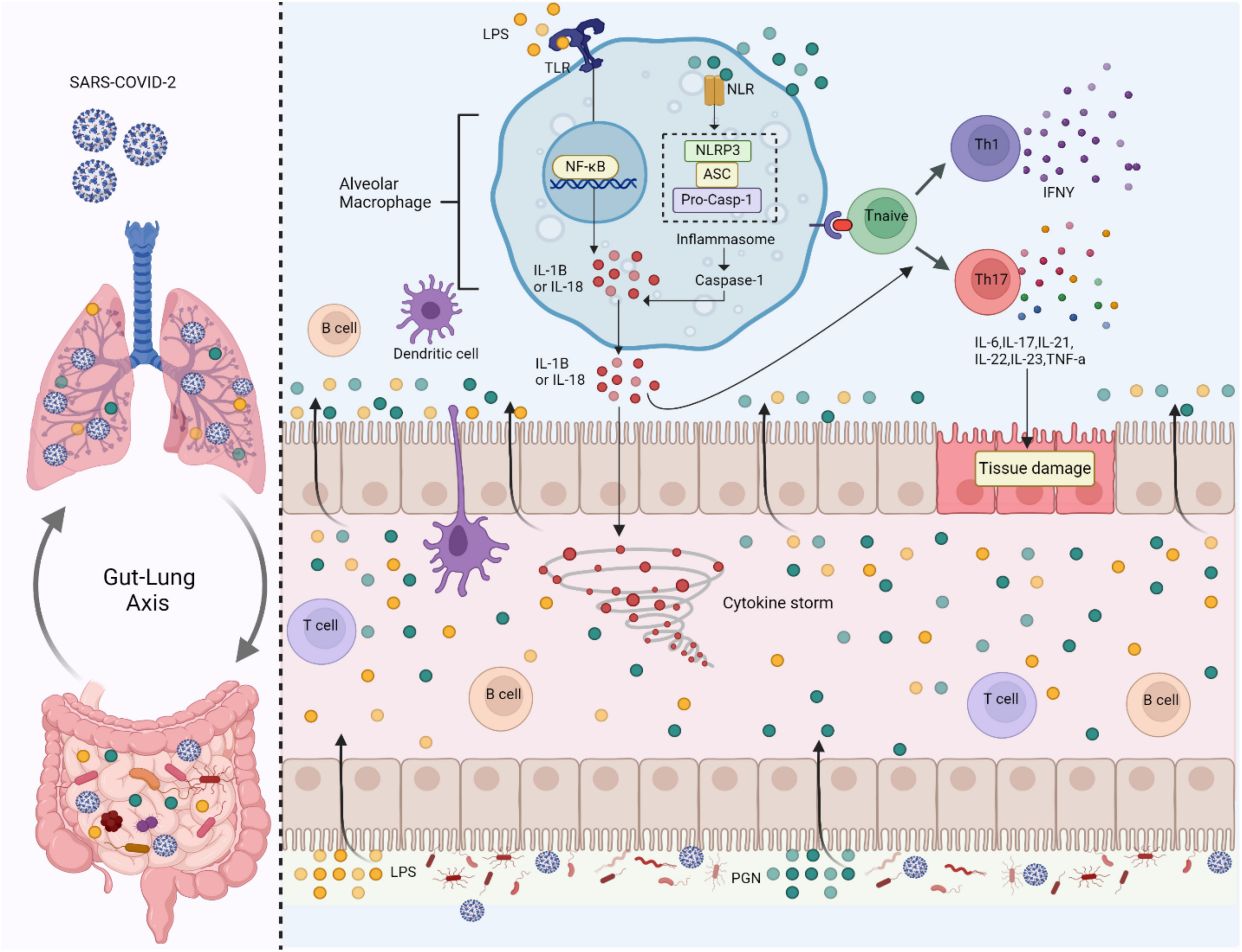
Gut microbiome imbalance disrupts the gut-lung axis. When SARS-CoV-2 infects the lungs, it activates Toll-like receptors (TLRs) on alveolar macrophages by recognizing microbial patterns such as Lipopolysaccharides (LPS) from pathogens. This triggers the Nuclear Factor Kappa- B Cells (NF-κB) signalling pathway, which promotes the release of pro-inflammatory cytokines, including Interleukin-1 Beta (IL-1β) and Interleukin-18 (IL-18). These cytokines drive the formation of the inflammasome (Nod-Like Receptor Family Pyrin Domain Containing 3 (NLRP3), ASC (Apoptosis-Associated Speck-Like Protein Containing a CARD) and Pro-Caspase-1), a protein complex that activates Caspase-1, leading to more IL-1β and IL-18 secretion and amplifying inflammation. In the gut, SARS-CoV-2-associated dysbiosis leads to an increase in pathogenic bacteria and bacterial components like Peptidoglycan (PGN) and LPS, which further activate TLRs. This induces inflammation in the gut epithelium and contributes to systemic immune activation. The cytokines and microbial products from the gut enter the circulation, exacerbating inflammation in the lungs and contributing to a cytokine storm, a life-threatening systemic inflammatory response. The immune response also involves T cells and B cells. Naïve T cells are polarized into T Helper Type 1 (Th1) and T Helper Type 17 (Th17) cells, releasing cytokines such as Interferon-Gamma (IFN-γ), Interleukin-6 (IL-6), Interleukin-17 (IL-17), Interleukin-21 (IL-21), Interleukin-22 (IL-22), Interleukin-23 (IL-23), and Tumor Necrosis Factor-Alpha (TNF-α). These cytokines cause tissue damage in both the gut and lungs, worsening the barrier function of the intestinal and pulmonary epithelia.

The lung microbiome is less examined than other microbiomes due to invasive sampling requirements and contamination risks. With increasing respiratory diseases, research into lung and gut microbiome interactions in disease pathogenesis is expanding (Aktas and Aslim, 2020). Chronic lung diseases cause microbial imbalances, favouring inflammation-inducing *Proteobacteria* due to altered lung conditions from persistent inflammation. Soluble metabolites such as peptidoglycan or LPS facilitate a communication network via the bloodstream between the lung and gut microbiota. This bidirectional interaction is often called the gut-lung axis (**Figure 4**) (Ancona et al., 2023). Additionally, existing literature suggests a correlation between the incidence of respiratory illnesses and the presence of chronic gastrointestinal ailments, including inflammatory bowel disease (Aktas and Aslim, 2020).

Respiratory infection by the influenza virus triggers systemic immune responses that lead to an increase of *Escherichia coli* in the gut, even without the virus’s presence there. On the other hand, Acetate from gut bacteria can activate interferon responses, offering protection against respiratory syncytial virus (Woodall et al., 2022). SARS-CoV-2 initially infects the pulmonary system, with potential subsequent systemic and multi-organ dissemination if pulmonary containment fails, underscoring the critical role of early gut microbiota modulation (Chen and Vitetta, 2022). Understanding the intricate and reciprocal influences between gut and lung microbiomes is currently limited; however, advancements in this field are anticipated to unlock new therapeutic avenues (Allali et al., 2021).

Under physiological conditions, gut-derived SCFAs enhance the function of immune cells in the lungs, such as macrophages and dendritic cells, fostering a balanced immune environment that prevents excessive inflammation (Kim et al., 2014; Wang et al., 2023). Conversely, cytokines and immune cells activated in the lungs during infection can affect gut microbiota composition, ensuring a coordinated systemic immune response​ (Belkaid and Hand, 2014). This systemic balance ensures robust defence mechanisms while preventing excessive inflammation.

The consequences of gut dysbiosis caused by SARS-CoV-2 are profound, with microbial translocation as a major driver of systemic inflammation. LPS and other bacterial endotoxins entering the bloodstream activate pattern recognition receptors such as TLRs on immune cells, triggering the release of pro-inflammatory cytokines (Ciesielska et al., 2020; Teixeira et al., 2021). This cascade culminates in a CS characterized by excessive IL-6, IL-1β, TNF-α, and IFNs, amplifying lung inflammation and increasing susceptibility to acute respiratory distress syndrome (ARDS)​ (Karki and Kanneganti, 2021; Vignesh et al., 2021). While IFNs (especially type I and III) are critical for antiviral responses, their dysregulated expression in SARS-CoV-2 infection paradoxically contributes to immune suppression and heightened inflammation (Galani et al., 2021). Moreover, dysbiosis-induced immune dysregulation impacts the pulmonary microbiome’s ability to maintain homeostasis, leading to aggravated viral replication and respiratory injury.

The CS significantly affects endothelial function and promotes coagulopathy, leading to widespread organ damage (Savla et al., 2021). Th17 cells, hyperactivated in the dysbiotic environment, exacerbate these systemic effects by sustaining inflammatory pathways, especially in severe cases (Huangfu et al., 2023). Elevated levels of IL-17 have been implicated in lung and vascular inflammation, linking gut dysbiosis to the respiratory and vascular complications observed in severe COVID-19, such as ARDS and thromboembolic events (Mikacenic et al., 2016; Mendoza, 2020; Hasan et al., 2021). Notably, the persistent inflammation driven by gut dysbiosis may also contribute to post-acute sequelae of COVID-19 (PASC), often referred to as long COVID, by maintaining a state of low-grade systemic inflammation​ (Raj et al., 2024). These perturbations in cytokines and over-activated pro-inflammatory cells perpetuate intestinal damage and disrupt pulmonary homeostasis, impairing the lung microbiome’s ability to prevent pathogenic colonization and maintain balanced immune responses and often leading to systemic inflammation​ (Kim and Shin, 2021). Several observational studies have demonstrated poor outcomes in COVID-19 infection related to systemic inflammation and immune dysregulation​ (Tang et al., 2020; Yeoh et al., 2021). The intricate interplay suggests that gut health is paramount in mitigating lung complications and systemic immune overactivation during SARS-CoV-2 infection. Understanding and modulating the gut-lung axis through interventions targeting microbiota may offer novel strategies for managing COVID-19.

#### 3.2.2 Gut-brain axis implications

**Figure 5:**
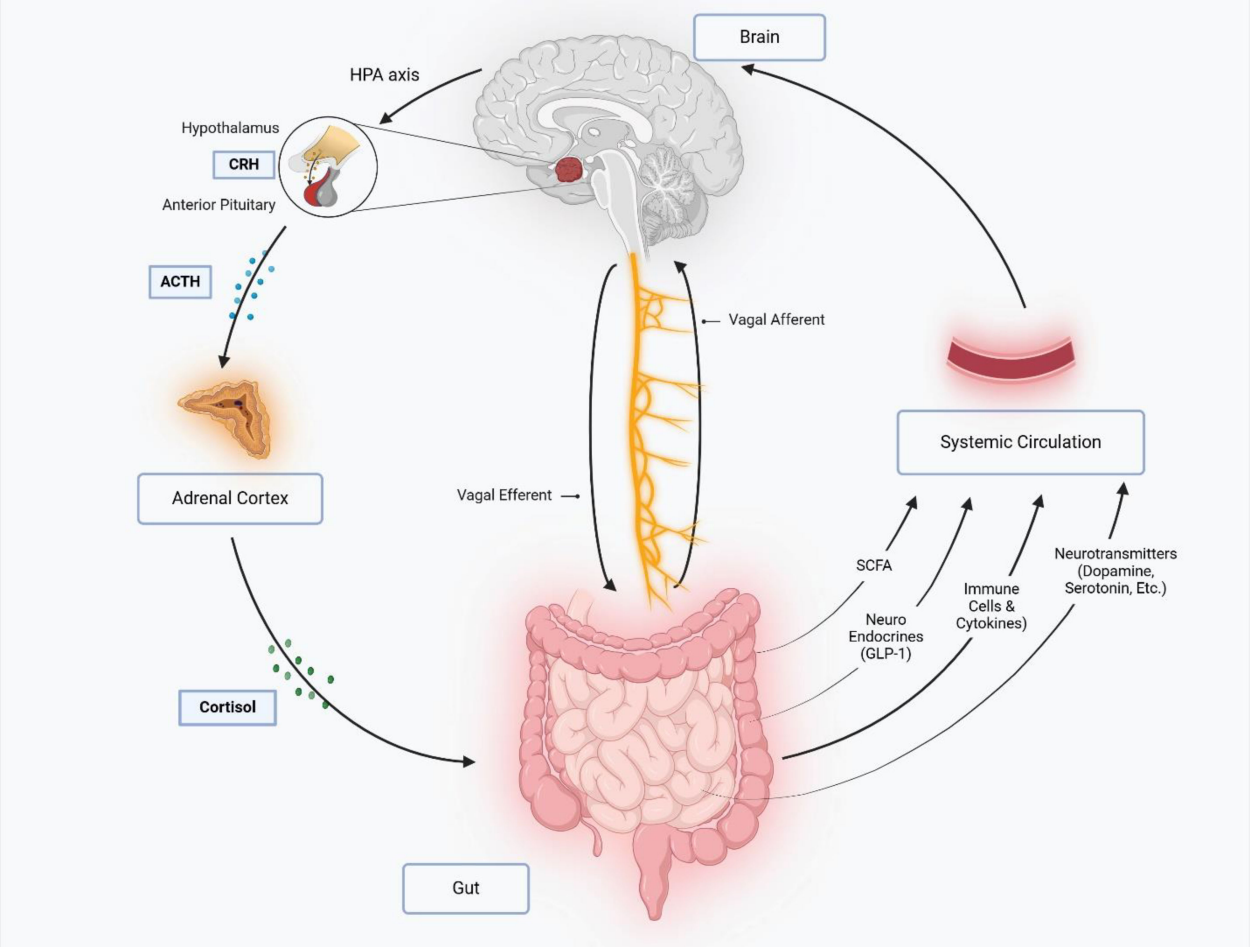
The gut-brain axis is a bidirectional communication network linking the central nervous system (CNS) and gastrointestinal tract via neural, endocrine, immune, and metabolic pathways. The vagus nerve mediates direct signaling through afferent (gut-to-brain) and efferent (brain-to-gut) fibers, while stress activates the hypothalamic-pituitary-adrenal (HPA) axis, triggering cortisol release that disrupts gut motility, barrier function, and immunity. Gut microbiota influence the gut-brain axis by producing short-chain fatty acids (SCFAs), neurotransmitters (e.g., serotonin, dopamine and GABA), and hormones like glucagon-like peptide-1 (GLP-1), which regulate neuro-inflammation, metabolism, and mood. Immune-derived cytokines from the gut can cross the blood-brain barrier, modulating neuro-inflammatory processes and cognition. This interplay integrates gut physiology with brain function, impacting stress responses, appetite, and mental health.

The gut-brain axis is a complex bidirectional communication network linking the GI tract and the central nervous system (CNS), mediated through neural, endocrine, immune, and metabolic pathways (**Figure 5**). Dysbiosis, or an imbalance in gut microbial communities, disrupts this system, leading to systemic inflammation and neurophysiological dysfunctions. Pro-inflammatory cytokines such as IL-6, TNF-α, and IL-1β, elevated in dysbiosis, can cross the blood-brain barrier (BBB), inducing neuroinflammation and contributing to neurodegenerative and psychiatric disorders (Yang et al., 2022; Zhang et al., 2023).

Dysbiosis also impairs the production of critical gut-derived metabolites, such as SCFAs, including butyrate, essential for anti-inflammatory signalling, gut barrier maintenance, and neuronal protection. Reduced SCFA levels during dysbiosis exacerbate inflammation and compromise the integrity of the gut-brain communication network (Johnson et al., 2021). Additionally, dysbiosis alters neurotransmitter synthesis, including serotonin, gamma amino butyric acid (GABA), and dopamine, affecting mood regulation and cognitive functions (Mhanna et al., 2023). These disruptions are implicated in conditions such as anxiety, depression, autism spectrum disorders (ASD), and neurodegenerative diseases like Alzheimer’s and Parkinson’s (Liu et al., 2020; Huang et al., 2021; Kalam and Balasubramaniam, 2024).

### 3.3 Metabolic syndromes, SARS-CoV-2 and gut dysbiosis

SARS-CoV-2-induced gut dysbiosis has profound implications for energy metabolism, particularly through the disruption of SCFA production and the exacerbation of systemic inflammation. SCFAs such as acetate, propionate, and butyrate, produced by the gut microbiota through dietary fibre fermentation, are essential for maintaining glucose and lipid homeostasis (Fusco et al., 2023). Butyrate, for instance, promotes the integrity of the gut epithelial barrier and has anti-inflammatory effects by inhibiting NF-κB signalling, while acetate and propionate enhance insulin sensitivity and regulate gluconeogenesis via the gut-liver and gut-brain axes (Wachsmuth et al., 2022; Korsten et al., 2023). Microbial diversity within the gut is significantly disrupted during SARS-CoV-2-mediated gut dysbiosis, with a marked reduction in SCFA-producing bacteria such as *Faecalibacterium prausnitzii* and *Roseburia* spp (Clerbaux et al., 2022). This reduction leads to impaired SCFA production and is linked to insulin resistance, increased adiposity, and systemic inflammation (Pham et al., 2023; Li et al., 2024).​

Propionate deficiency impacts hepatic gluconeogenesis regulation, causing prolonged glucose spikes, while acetate insufficiency interferes with fatty acid oxidation, increasing triglyceride accumulation and obesity risk (Shimizu et al., 2018; Liu et al., 2019; Yoshida et al., 2019; Aron-Wisnewsky et al., 2021). Research has demonstrated that individuals recovering from SARS-CoV-2 often show persistent metabolic disruptions, such as elevated blood glucose levels and dyslipidemia, partly due to these gut microbiota changes​ (Chen et al., 2023; Washirasaksiri et al., 2023). Addressing the metabolic consequences of SARS-CoV-2-mediated gut dysbiosis through targeted microbiota modulation and anti-inflammatory strategies holds promise for mitigating COVID-19 complications. In addition, it paves the way for enhancing resilience against future metabolic disorders.

### 3.4 Microbiome roles in long COVID-19 infection

Post-infection with COVID-19, while some individuals witness their gut microbiota stabilizing, others exhibit prolonged microbial disruption. Such sustained alterations in the gut’s microbial community have been detected for as long as six months following infection and may be implicated in the enduring symptomatology associated with long COVID-19 (Martín Giménez et al., 2023). Focus on COVID-19 has reduced over time, however, the issue of long COVID has surfaced as an important challenge for public health and economic stability worldwide (Álvarez-Santacruz et al., 2024).

Post-acute sequelae of COVID-19, widely recognized as long COVID, is a complex, multi-organ condition characterized by enduring and sometimes intense symptoms after the SARS-CoV-2 infection period, impacting nearly 65 million persons worldwide (Davis et al., 2023). The Centers for Disease Control and Prevention (CDC) identified the phenomenon of long COVID as a spectrum of symptoms that appear or persist over 28 days post-SARS-CoV-2 infection, with ongoing ambiguity in its comprehensive characterization and underlying mechanisms (Álvarez-Santacruz et al., 2024). A study indicated that a substantial portion of the cohort, exceeding one-third, manifested symptoms beyond the 10-week mark, meeting the criteria for progression to long COVID status. Another study indicated that Patients with long-term symptoms exhibited elevated levels of *Prevotella* and *Veillonella*, bacteria associated with inflammation due to LPS production.

The resemblance in the oral microbe composition between patients experiencing prolonged COVID-19 effects and those with chronic fatigue syndrome indicates a probable association between disruption in oral microbiota and the continual manifestation of long COVID symptoms. The referenced groups of bacteria are capable of translocating to the respiratory system by being inhaled from the mouth to the lungs, and they are associated with the initiation of various systemic ailments (Haran et al., 2021; Álvarez-Santacruz et al., 2024). Additionally, the research mentioned other species, the *Streptococcus anginosus* group, notable in respiratory infection pathogenesis, and *Gemella sanguinis*, implicated in bloodstream infections in COVID-19 patients, have been linked to long COVID pathology (Haran et al., 2021).

Long COVID is associated with gastrointestinal distress, like nausea, loss of appetite, diarrhoea, dysbiosis, and abdominal pain. SARS-CoV-2 antigenic proteins found in long COVID patients with inflammatory bowel disease indicate possible viral replication and persistent intestinal damage despite the futile attempts to culture the virus (Zhang et al., 2023). In a study involving recovered healthcare workers, persistent post-recovery symptoms have been associated with specific gut microbiota changes; higher levels of certain *Escherichia* species were linked to fatigue and myalgia, while *Intestinibacter bartlettii* was related to anorexia and fatigue. Conversely, increased counts of butyrate-producing species like *Intestinimonas butyriciproducens* and *Faecalibacterium prausnitzii* were inversely associated with chest tightness and cough (Zhou et al., 2021). Thus, the study suggested that gut microbiome imbalances may lead to ongoing COVID-19 symptoms post-recovery by influencing anti-inflammatory metabolite production like SCFAs and enabling opportunistic pathogens growth in patients. Despite the above findings, the exact mechanisms by which gut microbiota influence patient recovery necessitate further research (Zhou et al., 2021). Post-infection, sustained dysbiosis in the intestinal microbiota can induce neurological and respiratory pathologies via the gut-brain and gut-lung axes (Ancona et al., 2023). Intestinal microbes and their metabolites may influence lung health through direct migration and immune regulation. Furthermore, intestinal dysbiosis correlates with increased anxiety and depression in post-acute COVID-19 sequelae, suggesting a gut-brain axis involvement in mental health outcomes of long-COVID patients (Zhang et al., 2023). A continuous investigation is crucial to decipher the involvement of microbiota in the extended aftermath of COVID-19 in humans, as studies in animal models have revealed enduring modifications in the microbiota of the gut and lungs (Ancona et al., 2023).

## 4 Microbiome signatures as diagnostic biomarkers in COVID-19 detection

The gut microbiome’s integral role in shaping immunity and modulating inflammation suggests that its analysis may contribute to COVID-19 diagnostics (Farsi et al., 2022). In a cross-sectional analysis, bacterial diversity was notably diminished in COVID-19 patients relative to their healthy counterparts. The abundance of the Ruminococcaceae family and particular Lachnospiraceae genera (e.g., *Anaerostipes*, *Fusicatenibacter*, *Agathobacter*, *Eubacterium hallii*, and unclassified Lachnospiraceae) and *Roseburia* was notably decreased in the COVID-19 cohort. Such a decrease of *Roseburia*, a commensal bacterium producing the anti-inflammatory SCFA butyrate that supports mucosal barrier integrity and controls inflammation through mediators like IL-10, potentially exacerbating lung infection due to diminished butyrate-mediated regulation. On the contrary, the presence of *Streptococcus* (of Bacilli class) was increased in COVID-19 patients. Elevated *Streptococcus* abundance is a marker for opportunistic bacterial proliferation and is associated with an upregulation of proinflammatory cytokines, including IL-18, TNF-α, and IFN-γ, contributing to adverse clinical outcomes (Gu et al., 2020; Farsi et al., 2022). The same study selected a set of five bacterial biomarkers, including *Romboutsia*, *Actinomyces, Intestinibacter*, *Erysipelatoclostridium*, and *Fusicatenibacter*, to serve as distinguishing features separating COVID-19 cases from healthy controls (HCs) (Gu et al., 2020). The main findings in a pilot study conducted in Rome, Italy, included individuals with SARS-CoV-2 pneumonia, especially those in intensive care unit (ICU), showed marked divergence in microbial composition, with decreased richness, higher levels of *Proteobacteria*, and lower presence of *Spirochaetes* and *Fusobacteria*, in contrast to those not in ICU and controls (Mazzarelli et al., 2021). Further findings from the research are outlined in **Table 1**.

**Table 1:** Examples of microbiome diagnostic role.

Microbiome		Model	Sample	Cohort	Viral detection technique	Antibiotics/probiotics intake	The gut microbiota detection method	Reference
Enriched	Depleted							
COVID-19 cases compared to HCs: *Streptococcus* spp. (Bacilli class), in	COVID-19 cases compared to HCs: Lachnospiraceae family (*Eubacterium hallii*, *Fusicatenibacter* spp., *Agathobacter* spp., *Anaerostipes* spp., Unclassified Lachnospiraceae), *Roseburia* spp., Ruminococcaceae UCG-013	Human	Faecal	Total participants (n=84/males n=49, females n=35): (COVID-19 cases (n=30), hospitalized H1N1 cases (n=24), HCs having matched age, sex, and body mass index (n=30)). Mean age = 52.3	PCR	None	16S rRNA gene sequencing	(Gu et al., 2020)
Infectious disease wards patients compared to controls: Phylum level: *Proteobacteria* Family level: *Enterobacteriaceae*, *Actinobacteria*, *Staphylococcaceae*, *Peptostreptococcaceae*, *Vibrionaceae*, *Aerococcaceae*, and *Dermabacteraceae* ICU compared to controls: Family level: *Microbacteriaceae*, *Erysipelotrichaceae*, *Pseudonocardiaceae*, *Mycobacteriaceae*, and *Brevibacteriaceae* ICU compared to infectious disease wards: Family level: *Staphylococcaceae*, *Erysipelotrichales*, *Micrococcaceae*, *Microbacteriaceae*, and *Pseudonocardiaceae*	Infectious disease wards patients compared to controls: Phylum level: *Spirochaetes* and *Fusobacteria* Family level: *Nitrospiraceae*, *Moraxellaceae*, *Aeromonadaceae*, *Mycoplasmataceae*, and *Propionibacteriaceae* ICU compared to controls: Family level: *Carnobacteriaceae*, *Mycoplasmataceae*, and *Coriobacteriaceae* ICU compared to infectious disease wards: Family level: *Pectobacteriaceae*, *Coriobacteriaceae*, *Carnobacteriaceae*, *Selenomonadaceae*, *Moritellaceae*, and *Micromonosporaceae*	Human	Faecal	Total participants (n=23/COVID-19 cases n=15, negative controls n=8). Overall, patients with pneumonia were divided into 3 groups: ward COVID-19 patients over 18 years old, ICU COVID-19 patients over 18 years old, and negative cases for SARS-CoV-2 admitted to infectious disease wards and/or ICU	PCR	Two days at most, before rectal swab collection, 48% of subjects n=11 (5 infectious disease wards cases, 3 ICU cases, and 3 controls) followed antibiotic therapy.	16S rRNA gene sequencing	(Mazzarelli et al., 2021)
COVID-19 cases compared to HCs: Phylum level: Bacteroidetes (asymptomatic, mild, and severe), Proteobacteria (mild and severe), Actinobacteria (mild and severe) *Bifidobacterium* sp. (fivefold in severe groups) In COVID-19 patients: *Clostridium hathewayi*, *Ruminococcus gnavus*, *Parabacteroides distasonis*	COVID-19 cases compared to HCs: Phylum level: Firmicutes (asymptomatic, mild, severe) Family level: Lachnospiraceae (asymptomatic, mild, severe) Ruminococcaceae Totally absent in COVID-19 patients: *Butyricicoccus pullicaecorum*, *Lachnospira pectinoschiza*, *Pseudobutyrivibrio xylanivorans*, *Clostridium ruminantium*	Human	Faecal	Total participants (n=40/positive COVID-19 cases n=30, negative HCs n=10), the median age of COVID-19 cases ranging between 45.9-57.79 years old. Groups included (G1: HCs, G2: asymptomatic positive, G3: mild symptoms, G4: severe, oxygen-needed)	RT-PCR	Records of prior unsupervised medications not available	16S rRNA gene sequencing	(Khan et al., 2021)
Post-COVID-19 gut microbiota changes: *Bacteroides* and *Akkermansia*,	*Faecalibacterium, Roseburia*, and *Eubacterium.*	Human	Faecal	Total participants (n=149; 98 females, 51 males; aged 18-82 years). Classified into asymptomatic (n=10), mild (n=117), moderate (n=10), and severe (n=12). Antibiotic use: COVID-19 group (n=35) and COVID-19+ATB group (n=114). Controls (n=71; 51 females, 20 males, aged 18-79 years) were selected pre-pandemic.	RT-PCR	Some received antibiotics during acute illness	16S rRNA gene sequencing	(Ferreira-Junior et al., 2022)
COVID-19 cases compared to HCs: Opportunistic pathogens: *Enterococcus faecium*, *Klebsiella pneumoniae*	Beneficial bacteria: *Faecalibacterium prausnitzii, Bifidobacterium* spp.	Human	Faecal	Total participants (n=96; COVID-19 cases n=66, controls n=30)	RT-PCR	54% of COVID-19 patients received antibiotics	16S rRNA gene sequencing, metagenomic analysis	(Bernard-Raichon et al., 2022)
COVID-19 cases compared to HCs: Fungi: *Candida albicans* Bacteria: *Enterococcus, Lactobacillus*	Bacteria: *Faecalibacterium, Bacteroides*	Human	Fecal	Total participants (n=108): severe COVID-19 cases (n=40), mild COVID-19 cases (n=38), healthy controls (n=30); 10 severe cases were followed up approximately 6 months post-discharge.	RT-PCR	Not specified	16S rRNA gene sequencing for bacteria; ITS1 sequencing for fungi	(Maeda et al., 2022)
COVID-19 cases compared to HCs and recovered cases: Family: Prevotellaceae	Family: Bacteroidaceae, Ruminococcaceae, and Lachnospiraceae, Genus: *Faecalibacterium, Adlercreutzia* Species: *Eubacterium brachy* group	Human	Faecal	Total participants (n=60): COVID-19-positive patients (n=20), COVID-19-recovered individuals (n=20), and healthy controls (n=20).	RT-PCR	Antibiotic use was recorded and analyzed.	16S rRNA gene sequencing	(Yin et al., 2022)

ICU, intensive care unit; HCs, healthy controls; RT-PCR, reverse transcription polymerase chain reaction; PCR, polymerase chain reaction.

In addition, hierarchical clustering analysis revealed that the gut microbiota of COVID-19 ICU patients presented unique patterns not seen in non-ICU patients or controls (Mazzarelli et al., 2021). This study was limited by its small sample size, single-centre scope, possible ICU stay, and antibiotic impacts on gut microbiota (Mazzarelli et al., 2021). The intestinal microbiota and mucosal immunity are primary defence mechanisms against pathogenic intrusion. Higher faecal calprotectin in patients indicates a connection between gut inflammation and illness severity. Variations in the composition of the gut microbiome and the corresponding metabolic outputs contribute to metabolic inflammation, which is implicated in a range of health disorders (Khan et al., 2021). In a small, cross-sectional study, significant changes in the composition of bacteria were more evident in patients with severe COVID-19, with reductions in the Firmicutes/Bacteroidetes ratio present in cases of mild and severe illness (Khan et al., 2021). The study demonstrated that *Butyricicoccus pullicaecorum*, *Lachnospira pectinoschiza*, *Pseudobutyrivibrio xylanivorans*, and *Clostridium ruminantium* were absent in COVID-19 patients. At the same time, the abundance of the *Bifidobacterium* genus was elevated in cases of heightened severity (Khan et al., 2021). Additional microbial types are shown in **Table 1**.

In addition to the body’s microbiota, other factors can contribute to diagnosing COVID-19. In a study analyzing the metabolome in faecal samples of COVID-19 patients, changes in faecal metabolite profiles shed light on gut metabolism and absorption, playing a key role in elucidating the pathophysiology of diseases, pinpointing diagnostic markers, and enabling early detection and prognostication of disease development (Lv et al., 2021). Findings included certain metabolites detected in faecal samples, such as ribonic acid, pseudouridine, sucrose, D-allose, and 7H-purine, having importance in distinguishing the metabolomic profiling of COVID-19 cases compared to HCs. In severe COVID-19 cases, there was an increase in urea, lactic acid, and cyclohexanecarboxylic acid levels. In contrast, deoxycholic acid, monomethyl succinate, 1-pentadecanol, D-cellobiose, and propanoic acid were found at lower levels than in mild cases (Lv et al., 2021). The same study demonstrated that in COVID-19 patients, certain correlations between faecal metabolites and microbial populations diverge from the patterns in HCs, suggesting disease-specific alterations. For instance, elevated 1,5-anhydroglucitol levels in COVID-19 patients were inversely associated with *Streptococcus* bacterial growth. Another example is a reduction in D-arabinose associated with a decline in *Aspergillus rugulosus* (Lv et al., 2021).

## 5 Microbiome content as a prognostic indicator for COVID-19 outcomes

Specific microbial abundances in the human microbiome serve as biomarkers to differentiate individuals with COVID-19 from those who are healthy (Reuben et al., 2023). Shifts in the gut’s microbial makeup are being identified as influential predictors for determining the severity and likely outcomes of COVID-19 infection, highlighting the importance of shaping the bodily immune responses and impacting how the illness unfolds. Variations in the microbes in the upper respiratory passages when SARS-CoV-2 infection starts have been correlated with the potential to anticipate the chances of a patient’s survival (Wang et al., 2023; Zhou et al., 2023). Several studies had a consensus on the diversity shifts within the gut microbiota, notably in *Faecalibacterium*, *Bacteroides*, *Roseburia*, *Lactobacillus*, *Eubacterium*, *Ruminococcus*, *Clostridium*, and *Bifidobacterium*, which have been connected to both the clinical developments and prognostic assessments of COVID-19 (Farsi et al., 2022). The elderly are at greater risk for severe COVID-19 outcomes. Gastrointestinal disturbances may precede COVID-19 symptoms and correlate with gut microbial dysbiosis. Specifically, the reduction in butyrate-producing bacteria has been related to adverse effects in critically ill patients, while elevated levels of *Actinobacteria* may suggest a negative prognosis. Hence, gut microbiota profiles may hold predictive value for the severity of COVID-19 (Qiu et al., 2024). Dysbiosis paves the way for opportunistic pathogens like *Enterococcus* to thrive. Disrupted gut barriers could enable enterococcal translocation into the bloodstream, posing further health risks in COVID-19 patients. Understanding the coexistence and impact of SARS-CoV-2 and *Enterococcus* on disease severity is necessary for clinical outcomes (Giacobbe et al., 2021; Wang et al., 2023).

## 6 Microbiome-driven approaches for the improvement of COVID-19 outcomes

**Figure 6:**
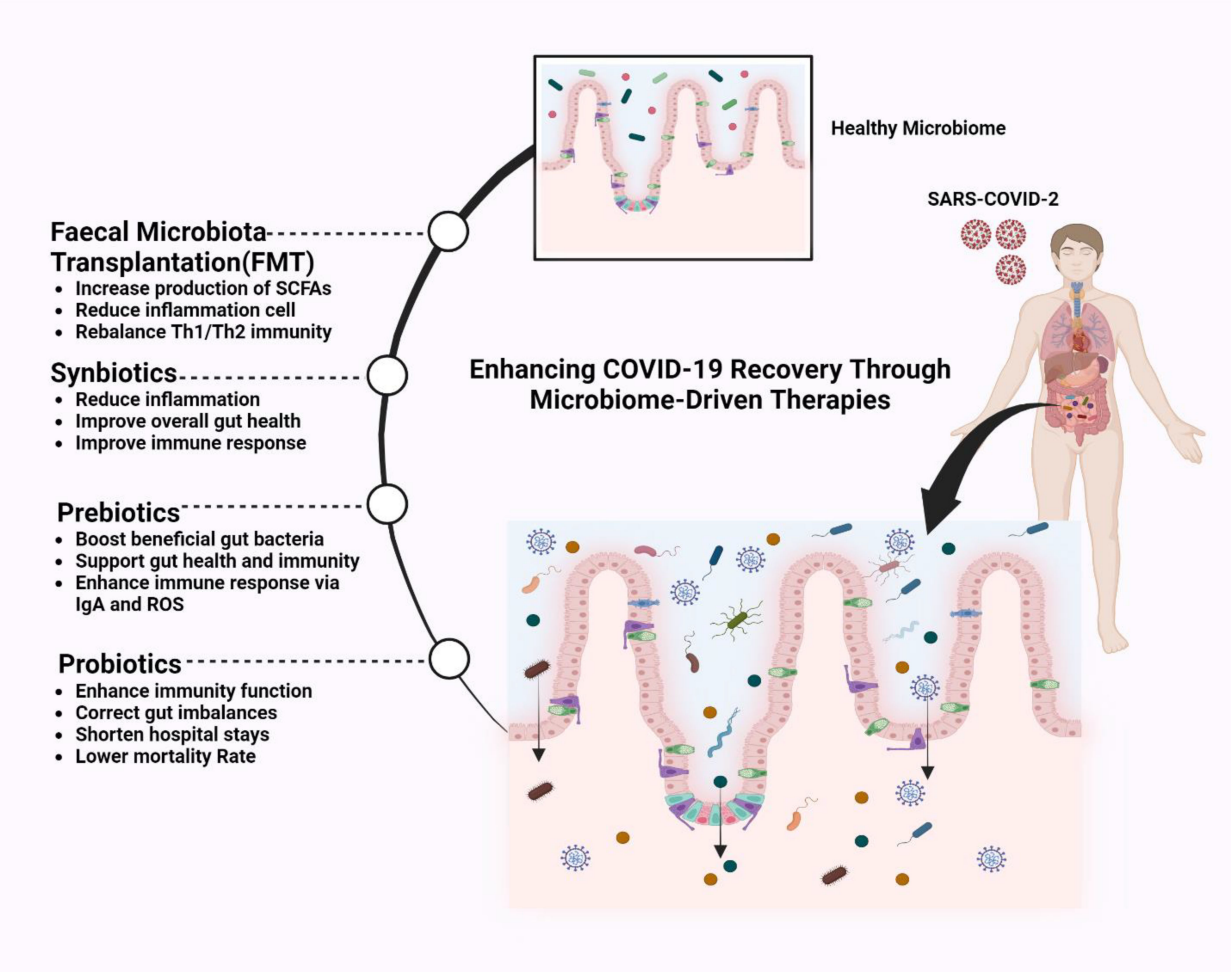
Improving COVID-19 management by enhancing the gut microbiome. Microbiome-driven therapies to enhance COVID-19 recovery, including faecal microbiota transplantation (FMT), which restores gut balance, increases SCFAs (Short-Chain Fatty Acids), reduces inflammation, and rebalances T-helper cell type 1 (Th1) and T-helper cell type 2 (Th2) immunity. Synbiotics (probiotics and prebiotics) and Prebiotics boost beneficial bacteria, improve immunity via immunoglobulin A (IgA) and Reactive Oxygen Species (ROS), and reduce inflammation. Probiotics correct gut imbalances, enhance immunity, and lower mortality rates. These therapies strengthen gut health, supporting immune function and recovery in COVID-19 patients.

Therapeutic strategies for COVID-19 might encompass modulating gut microbiota to enhance patients’ health. This can involve administering probiotics, prebiotics and postbiotics to augment beneficial bacterial populations and employing prebiotics to stimulate their growth, thereby generating advantageous metabolites. Faecal microbiota transplantation (FMT) involves transferring favourable microbial communities from a healthy donor to persons afflicted by a COVID-19 infection. These approaches aim to resolve dysbiosis and possibly contribute to improved clinical outcomes in COVID-19 cases (**Figure 6**).

### 6.1 Probiotics and prebiotics

Variations in gut microbial composition and their metabolic activities could potentially modify the effectiveness of vaccines. Research indicates that microbiome adjustment post-vaccination might alter immunization outcomes. Therefore, approaches targeting gut microbiome modification could be utilized to amplify vaccine potency. Furthermore, data implies that COVID-19 vaccination might impact the variability within the gut microbial community (Qiu et al., 2024). Probiotics, like bifidobacteria and lactobacilli, may strengthen immune function and reduce COVID-19 symptoms by balancing gut microbiota, particularly in older patients with virus-induced gastrointestinal complications. They could also mitigate gastrointestinal side effects related to COVID-19 treatment, highlighting the importance of the gut-brain axis in COVID-19 management (Qiu et al., 2024).

Live microorganisms (probiotics) and substances that feed beneficial gut bacteria (prebiotics) show promise as additional COVID-19 treatments. Specific probiotic strains, such as *Lactobacillus rhamnosus* GG, *Bifidobacterium lactis*, and *Lactobacillus plantarum*, have demonstrated antiviral and immunomodulatory effects. For instance, *Lactobacillus plantarum* DR7 has been shown to suppress plasma proinflammatory cytokines, aiding in the management of upper respiratory tract viral infections (Takiishi et al., 2017). Additionally, *Lactobacillus* species produce metabolites like bacteriocins and lactic acid, which have antiviral properties. These metabolites can inhibit viral replication and protect against infections (Kiela and Ghishan, 2016). Furthermore, metabolites from *Lactobacillus plantarum* can bind to ACE2 receptors, potentially blocking SARS-CoV-2 attachment to host cells (Taufer et al., 2024).

The review done by Rabei et al. evaluates the role of probiotics in mitigating COVID-19 outcomes, synthesizing evidence from clinical trials conducted between 2020 and 2023 (Rabiei et al., 2024). The authors highlight that probiotic supplementation—particularly strains of *Bifidobacterium* (e.g., *B. lactis*, *B. longum*) and *Lactobacillus* (e.g., *L. rhamnosus*, *L. plantarum*) reduces key symptoms such as fever, cough, diarrhoea, and nasal congestion, accelerates recovery, and lowers mortality rates by modulating immune responses, enhancing gut barrier integrity, and suppressing cytokine storms via mechanisms like SCFA production. Clinical trials demonstrate that probiotics decrease viral shedding, inflammatory markers (e.g., IL-6, CRP), and reliance on mechanical ventilation while improving antibody responses (IgM/IgG) and post-acute COVID-19 syndrome outcomes. The gut-lung axis and probiotic-mediated immune regulation (e.g., T-cell activation, NF-κB inhibition) are central to these benefits. The randomized controlled trial (RCT) by d’Ettorre et al. (2020) administered a combination of *Lactobacillus paracasei*, *L. plantarum*, and *Bifidobacterium lactis* to hospitalized COVID-19 patients, resulting in a 60% reduction in diarrhoea and a 50% decrease in nasopharyngeal viral load compared to placebo (d’Ettorre et al., 2020).

Nutritional changes can affect gut microbiota and possibly improve outcomes in COVID-19, with research linking diet to variations in disease severity and mortality (Zhang et al., 2023). In a study done with elderly care facility inhabitants during the pandemic of COVID-19 where the probiotic strain *Ligilactobacillus salivarius* MP101 was administered as dairy products, results showed that the strain could foster health in at-risk persons. Furthermore, this probiotic treatment significantly affected the concentrations of inflammatory mediators, specifically IL-8 and IL-19 (Mozota et al., 2021). Meta-analysis findings proposed that the adjunctive use of probiotics in COVID-19 management may facilitate a reduction in hospitalization duration, accelerate healing, and diminish mortality probabilities. Yet they are not effective as preventative measures against COVID-19 (Sohail et al., 2023). Strains of lactic acid bacteria may potentiate antiviral defences, potentially restricting viral access and replication. In addition, certain bacteria may also stimulate the production of antibodies to target the virus (Qiu et al., 2024). In a study, it was concluded that the supplementation of *Lactiplantibacillus plantarum* HEAL9, a strain used as a probiotic strategy, has the potential to alleviate neuroinflammation-related symptoms, including mood, sleep and cognitive complications post-COVID-19 by modifying the gut-brain axis (Önning et al., 2023; Qiu et al., 2024).

The prebiotic role comes into play after consuming fibres from plant origin, which fosters the growth of favourable gut microbiota alongside the reduction of pathogenic ones like Clostridia. The fermentation of these fibres by the beneficial microbes leads to the generation of SCFAs, which are vital for sustaining the health of the intestinal barrier and modulating immune function (Hussain et al., 2021). In both animal and human models, studies proposed that prebiotics modulate immunity, specifically by the amplification of secretory IgA and enhancing the production of ROS (Antony et al., 2023). Prebiotics like inulin and galactooligosaccharides (GOS) also show promise by stimulating the growth of SCFA-producing bacteria, such as *Faecalibacterium prausnitzii*, which downregulate pro-inflammatory cytokines (IL-6, TNF-α) via histone deacetylase inhibition (Donkers et al., 2024). Synbiotics, combinations of probiotics and prebiotics, are being tested in clinical trials (e.g., NCT04813718) to amplify these effects.

Synbiotics, a synergistic combination of pro- and pre-biotics, could improve health and attenuate inflammation, as evidenced by the reductions in proinflammatory leukocytes and the cytokine IL-6 (Antony et al., 2023). A 2024 Lancet Infectious Diseases study by Vaezi and Ravanshad showed if SIM01, a synbiotic, helped post-acute COVID-19 syndrome using a randomized, double-blind, placebo-controlled trial. The study involved 464 participants in Hong Kong with persistent symptoms (e.g., fatigue, memory loss, gastrointestinal upset) following acute COVID-19. Results showed that after six months, the SIM01 group had significantly higher rates of symptom alleviation compared to placebo, including reduced fatigue (OR 2.27), improved memory (OR 1.97), better concentration (OR 2.64), and resolved gastrointestinal issues (OR 1.99). The synbiotic also enhanced gut microbiota diversity and reduced inflammatory markers, suggesting gut microbiome modulation as a key mechanism for mitigating long COVID symptoms. These findings highlight SIM01 as a promising therapeutic strategy for improving recovery in long COVID patients through microbiome-targeted intervention (Vaezi et al., 2023).

### 6.2 Postbiotics

Postbiotics, the bioactive compounds derived from probiotics, have gained significant attention as potential adjuvant therapies for SARS-CoV-2 infection. These metabolites, including bacteriocins, exopolysaccharides (EPS), and SCFAs, exhibit antiviral and immunomodulatory properties. Studies suggest that postbiotics can modulate gut microbiota, enhance immune responses, and reduce inflammation by inhibiting ACE2 activity, the primary receptor for SARS-CoV-2 entry. Additionally, postbiotics have been found to stimulate regulatory T cells, reducing pro-inflammatory cytokines such as IL-6 and TNF-α, which play a role in the severity of COVID-19-related cytokine storms (Khani et al., 2022)​.

Furthermore, postbiotics demonstrate direct antiviral effects by blocking viral attachment to host cells and enhancing mucosal immunity. Compounds such as *Lactobacillus*-derived metabolites have shown potential in disrupting the SARS-CoV-2 envelope and inhibiting viral replication. Their safety, stability, and long shelf life make postbiotics an attractive alternative to traditional probiotics, particularly in patients with compromised immune systems. The gut-lung axis also plays a critical role in immune homeostasis, with postbiotics influencing systemic immune responses beyond the gastrointestinal tract. Given these promising findings, further clinical research is needed to explore the full potential of postbiotics in preventing and managing viral infections, including COVID-19 (Khani et al., 2022).

### 6.3 Fecal microbiota transplantation

FMT involves transferring faecal matter from a healthy donor to restore gut microbial balance. In a landmark case series. The efficacy of FMT and probiotics is speculative, requiring forward-looking studies to understand the mechanisms involved (Zhang et al., 2023). Study findings indicated that FMT can replenish beneficial gut bacteria, increasing SCFAs. FMT aided in rebalancing Th1/Th2 immunity and reduced immune components such as mast cells, basophils, IgE, and eosinophils (Kim et al., 2021).

The review by Kazemian et al. (2021) explores the implications of FMT during and post-COVID-19, emphasizing the intricate relationship between gut microbiota and SARS-CoV-2 pathogenesis. The authors highlight the gut–lung axis, suggesting that microbial metabolites like SCFAs and bile acids play a critical role in immune regulation and may influence the effectiveness of FMT. The paper underscores the need for stringent donor screening protocols to mitigate potential risks associated with asymptomatic viral transmission through FMT. Given the documented alterations in gut microbiota composition among COVID-19 patients, the authors stress the importance of investigating whether FMT can help restore microbial diversity and enhance immune resilience in recovering patients (Kazemian et al., 2021).

A study by Liu et al. administered ten oral FMT capsules over four days to patients, observing notable improvements in gastrointestinal symptoms such as diarrhoea, constipation, abdominal pain, and acid reflux alongside psychological benefits like reduced fatigue, depression, and insomnia. Microbiome analysis revealed a significant reduction in Proteobacteria and increases in beneficial taxa such as *Actinobacteria, Bifidobacterium, Faecalibacterium*, and *Collinsella* post-FMT. Pre-treatment, dominant genera included *Bacteroides* (28.3%), *Prevotella* (13.0%), and *Faecalibacterium* (6.5%). Post-FMT, *Bacteroides* remained prevalent (31.1%), with marked rises in *Bifidobacterium* (10.4%) and *Faecalibacterium* (11.7%). Immunologically, FMT increased naïve B cells, immature regulatory B cells, and double-positive T cells while reducing total B cells. However, no significant changes were detected in erythrocyte counts, liver enzymes (ALT/AST), kidney function markers, or NK/T-cell populations (Liu et al., 2021).

## 7 Challenges and future directions in microbiome-driven COVID-19 therapies

The application of microbiome-based therapies in COVID-19 management faces significant challenges due to interindividual variability in gut microbiota composition, genetic factors, and underlying comorbidities (Valles-Colomer et al., 2022). For example, individuals with Prevotella-dominant gut microbiomes may exhibit enhanced responses to fibre-based interventions compared to those with Bacteroides-dominant profiles, necessitating a personalized approach to microbiome modulation (Hjorth et al., 2020; Gibbons et al., 2022). Furthermore, despite growing interest in probiotics, prebiotics, and faecal microbiota transplantation (FMT) for modulating immune responses and alleviating post-viral complications, substantial evidence gaps remain. Most existing studies are observational or limited in sample size, underscoring the need for large-scale RCTs to validate efficacy. Ongoing trials, such as NCT04950803, are currently investigating the potential of synbiotics in mitigating long COVID symptoms, but more rigorous research is required to establish clinical guidelines (Lau et al., 2024).

Safety remains a critical concern in microbiome-targeted interventions, particularly for immunocompromised individuals. While probiotics have demonstrated benefits in reducing inflammation and promoting gut-lung axis homeostasis, they also pose risks, such as probiotic-associated bacteremia or fungemia in vulnerable populations (Suez et al., 2018). Similarly, FMT has been explored as a means of restoring gut microbial balance post-COVID-19, yet it carries the risk of transferring antibiotic-resistant genes or pathogenic microorganisms if donor screening is inadequate (Kazemian et al., 2021). As microbiome-based therapeutics advance, stringent safety protocols, standardized dosing regimens, and comprehensive screening measures will be crucial in ensuring both efficacy and safety.

The future of microbiome-driven COVID-19 therapies lies in personalized, data-driven interventions that integrate cutting-edge technologies. Machine learning algorithms hold promise for analyzing complex metagenomic, metabolomic, and clinical datasets to generate individualized recommendations for probiotic supplementation or dietary modifications (Putignani et al., 2019). Such approaches could optimize treatment responses based on an individual’s unique microbial signature, increasing therapeutic precision and effectiveness. Additionally, the development of postbiotics—bioactive, non-viable microbial components such as bacterial lysates, exopolysaccharides, and short-chain fatty acids—offers a safer alternative to live probiotics, particularly in immunocompromised patients (Ma et al., 2023). Postbiotics have been shown to exert immunomodulatory effects, modulating cytokine responses and enhancing gut barrier function without the risks associated with live bacterial administration.

Another emerging avenue is the integration of multi-omics approaches, including viromics, mycobiomics, and resistomics, to gain a holistic understanding of the gut-lung axis and its role in COVID-19 pathophysiology (Eshetie et al., 2024). By unravelling complex host-microbe interactions, these comprehensive methodologies could lead to highly targeted interventions that address microbial dysbiosis, immune dysfunction, and systemic inflammation in COVID-19 patients. As microbiome research advances, collaborative efforts between microbiologists, immunologists, and data scientists will be essential in translating these discoveries into clinically viable treatments.

## 8 Conclusion

SARS-CoV-2 disrupts the gut microbiome, causing dysbiosis that significantly affects COVID-19, impacting the immune system, reducing SCFA production, disrupting communication between the gut and the brain and lungs, and compromising intestinal barrier function. Disease severity is associated with an imbalance in microbes, marked by fewer beneficial species and more inflammatory ones. Probiotics and prebiotics offer therapeutic benefits, which restore microbial balance, improve immunity, and reduce inflammation. The profile of gut microbes provides valuable information for diagnosing and predicting disease progression, emphasizing their role in treatment decisions.
